# Flexible Search for Single-Axon Morphology during Neuronal Spontaneous Polarization

**DOI:** 10.1371/journal.pone.0019034

**Published:** 2011-04-29

**Authors:** Honda Naoki, Shinichi Nakamuta, Kozo Kaibuchi, Shin Ishii

**Affiliations:** 1 Graduate School of Informatics, Kyoto University, Uji, Kyoto, Japan; 2 Department of Cell Pharmacology, Nagoya University, Nagoya, Aichi, Japan; 3 RIKEN Computational Science Research Program, Wako, Saitama, Japan; Rikagaku Kenkyūsho Center for Allergy and Immunology, Japan

## Abstract

Polarization, a disruption of symmetry in cellular morphology, occurs spontaneously, even in symmetrical extracellular conditions. This process is regulated by intracellular chemical reactions and the active transport of proteins and it is accompanied by cellular morphological changes. To elucidate the general principles underlying polarization, we focused on developing neurons. Neuronal polarity is stably established; a neuron initially has several neurites of similar length, but only one elongates and is selected to develop into an axon. Polarization is flexibly controlled; when multiple neurites are selected, the selection is eventually reduced to yield a single axon. What is the system by which morphological information is decoded differently based on the presence of a single or multiple axons? How are stability and flexibility achieved? To answer these questions, we constructed a biophysical model with the active transport of proteins that regulate neurite growth. Our mathematical analysis and computer simulation revealed that, as neurites elongate, transported factors accumulate in the growth cone but are degraded during retrograde diffusion to the soma. Such a system effectively works as local activation-global inhibition mechanism, resulting in both stability and flexibility. Our model shows good accordance with a number of experimental observations.

## Introduction

Establishing polarity in cells is an essential step for many biological functions. Cellular polarization is regulated by intracellular chemical reactions and by active protein transport mediated by motor proteins, and it is accompanied by cellular morphological changes regulated by cytoskeletal rearrangements [1], [2], [3]. To elucidate the general principles underlying polarization, we focused on developing neurons as a model system. A developing neuron requires a typical polarized morphology, consisting of a soma, an axon and dendrites, for the unidirectional transfer of electric signals between neurons.

**Figure 1:**
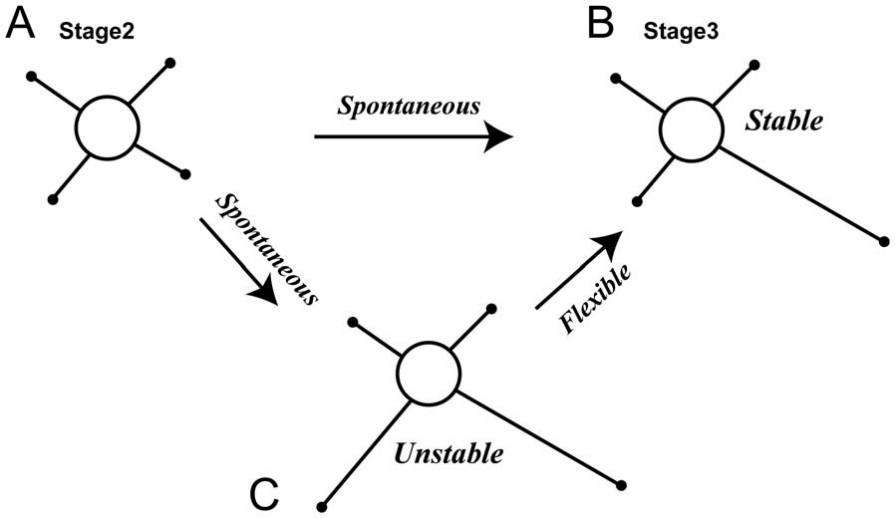
Flexible search for single-axon morphology. (**A**) In stage 2, a neuron has several short neurites of similar length. (**B**) A developing neuron enters stage 3 if one neurite spontaneously elongates and develops to an axon. This established polarity is stably maintained. (**C**) Sometimes multiple neurites accidentally elongate, but this unfavorable pattern is flexibly reversed, leading to the single-axon pattern (B).

The morphological processes of neurons have been divided into five stages [4]. In stage 1, a cell first produces many thin extensions called filopodia. During stage 2, several filopodia are stabilized as short neurites of similar length (**Figure 1A**). After several hours, one neurite abruptly begins to elongate and obtains the properties of an axon in stage 3, which corresponds to polarization (**Figure 1B**). During these stages, various molecules involved in neurite extension are transported by motor proteins from the soma to the neurite tips called growth cones which are responsible for neurite motility [5], [6], [7], [8], [9], [10]. Several days after polarization, other neurites acquire dendritic characteristics and begin to form premature synapses during stages 4 and 5.

Neuronal polarization occurs in spite of uniform symmetrical distributions of extracellular chemical and adhesion factors [4], [11], indicating that developing neurons have the ability to spontaneously disrupt their own morphological symmetry. To achieve polarization, the elongation of a selected neurite must be spontaneously induced and accompanied by the suppression of the other neurites so that they do not extend further (**Figure 1B**). How is such a *stable,* winner-takes-all mechanism achieved under symmetrical conditions? Sometimes multiple neurites accidentally elongate during the polarization process (**Figure 1C**), but this unfavorable morphological pattern is eventually reversed so that a single axon remains, as though the cell is seeking a more favorable morphological pattern. How is such a *flexible* mechanism for searching single-axon morphology achieved?

Polarized pattern formation has been examined extensively, with particular attention to reaction-diffusion systems with a local activation-global inhibition mechanism [12], [13]. However, cellular polarization does not strictly adhere to the reaction-diffusion framework; the molecules that mediate global inhibition have not been experimentally identified [2]. Polarization involves the active transport of various molecules and dynamic changes in morphology in addition to the reaction-diffusion system. Questions remain as to whether a local activation-global inhibition mechanism is plausible as a complete system without inhibitor molecules.

Theoretical studies seeking to address this question have modeled neuronal polarization with the active transport of a neurite elongation-promoting factor [14], [15], [16]. In two of these studies [14], [15], the rate of transport from the soma was assumed to be enhanced by the concentration of some molecules at the growth cone. These assumptions introduced local activation; active transport is selectively limited to a single neurite (i.e., the axon candidate), leaving the other neurites unpolarized. It is puzzling, however, how the soma (or active transport system working in the soma) knows the state (variable values) of each growth cone. Several existing studies assumed that a specific transported molecule is under mass-conservation without continuous production and degradation [15], [16], whereas protein degradation is known to be involved in polarization [17]. The mass-conservation assumption directly introduced global inhibition, due to the competition of the mass-conserved molecule between all growth cones through active transports. Hence, the existing models have successfully induced spontaneous polarization, highly relying on the assumptions of selective active transport to a winner neurite and/or conserved pool of transported molecule. In all of these models, moreover, growth rate of neurites was assumed to be monotonically increasing against the concentration of transported molecules at the growth cone [14], [15], [16], although experimental studies have observed axonal neurite suddenly elongates in a switch-like manner [4]; fluorescence resonance energy transfer (FRET) imaging has also shown that a small GTPase, H-Ras, is significantly activated only in the axonal growth cone and not in the shorter neurites [15], which is an another evidence for the involvement of switching molecules. Then, these existing models cannot fully explain the mechanism of flexible search for single-axon morphology from an unfavorable state of multiple long neurites, probably due to the lack of the nonlinear switch regulating the growth cone.

Building on these earlier studies, we introduce both degradation of the transported molecule and a bistable switch for axon specification at each growth cone, which is activated by the transported molecule. Our mathematical analysis revealed the logic of a local activation-global inhibition mechanism that is based on active transport, degradation and morphological changes (neurite growth), without the need for global inhibitor molecules. Based on this analysis, we presented the mechanism of flexible search for single-axon morphology during neuronal polarization, which was confirmed by computer simulations. Furthermore, we found that the introduction of a bistable axon specification allows our model to reproduce many experimental observations.

## Results

### Model for Active transport, diffusion and degradation of factor X

**Figure 2:**
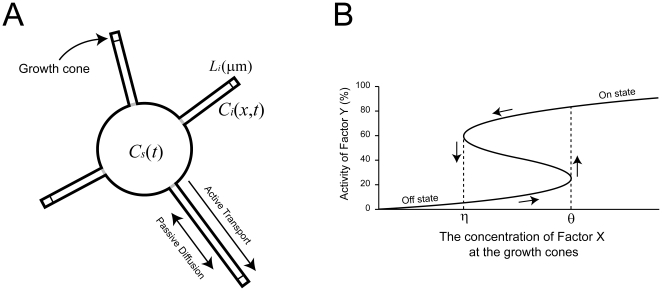
Model of neuronal polarization. (**A**) The model neuron consists of one soma, a well-mixed compartment, and several neurites, each of which is represented as a continuous cable compartment. Each neurite has a growth cone at the tip. Factor X is produced by gene expression in the soma and is actively transported to each growth cone. Factor X is degraded throughout the cell and diffuses along the neurite shafts. 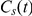 denotes the concentration of factor X in the soma at time *t*, and 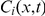 denotes the concentration of factor X at *x* µm from the neck of neurite *i* at time *t*. 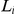 is the length of neurite *i*. (**B**) The axon is specified according to the activity of factor Y at the growth cone. Factor Y activity depends on the concentration of factor X but also has hysteresial behavior. Between the threshold concentrations of *η* and *θ*, the system exhibits bistability with one unstable and two stable states (on and off states). When factor Y is in the on state, the neurite elongates, and when factor Y is in the off state, the neurite shrinks.

The model neuron consists of *N*+1 compartments: one soma and *N* neurites (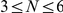) (**Figure 2A**). The somatic cytoplasm was modeled as a well-mixed compartment with a volume of *V*, and each neurite shaft was considered to be a continuous compartment with a cross-sectional area *A* and length *L_i_*, where the subscript *i* indexes a neurite. Factor X is involved as a key regulator of axon specification and is produced by gene expression in the soma at rate *G*. Factor X is degraded (or inactivated) at rate *k*; it diffuses at rate *D* throughout the entire neuron and is transported stochastically by motor proteins and/or waves that depend on actin filaments [18], [19] from the soma to each growth cone. This transport is assumed to be Poissonian, and the rate is proportional to the somatic concentration of factor X. No time delay in transport is considered in the model because the time required for the motor proteins to travel to the growth cone is negligible in comparison to the time scale of polarization (∼36 h); the kinesin motor protein moves with a velocity of 0.2 - 1.5 µm/s along neurites, which are approximately 10 µm long [4], [20]. The dynamics of the concentration of factor X in the soma, 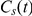, and along each neurite, 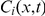, are described as:
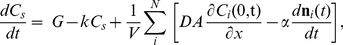
(1)

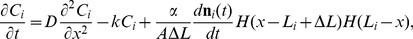
(2)with boundary conditions 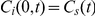, where *x* represents the distance from the neck of each neurite and α is a positive constant representing the amount of factor X per single transport. The variable 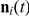 designates the number of active transport events to each neurite tip at time *t* and is an independent random variable sampled from a Poisson probability distribution with the frequency 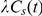. With each transport event, factor X is released at the tip of a neurite of length 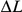. 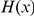 is a step function in which 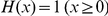 or 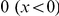. Initially, the concentration of factor X is 0 in the entire cell, and the length of every neurite is *L_o_*. The diffusion and degradation of proteins are usually fast and slow, respectively; the proteins in the neurons disperse with diffusion coefficients of 0.23–44 µm^2^/s [21], [22] and they have a half-life of approximately one day [16], [23].

### Model for regulation of neurite growth by factor Y

Axon specification in stage 2, during which one of the neurites extends suddenly, is considered to be a threshold phenomenon, and its stable nature suggests the existence of bistability. In the model, we assume that a certain molecule, factor Y, plays an essential role in axon specification at the growth cones. Factor Y is activated by factor X, but its activation is characterized by hysteresis (**Figure 2B**), implying that factor Y works as a bistable switch. Hysteresis is observed in many dynamic systems with nonlinear feedback loops. Feedback loops have been found in growth cone signaling [2]. A small GTPase is activated in only one growth cone, in accordance with axon specification [15]. We have developed a potential model of a reaction network that produces a bifurcation diagram exhibiting hysteresis (**Figure S1**). Our model assumes that if factor Y is in an ‘on’ (or an ‘off’) state, the neurite elongates (or shrinks to *L_o_*) as follows: 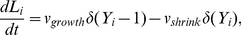
(3)where 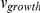 and 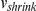 are the rates of growth and shrinkage of the growth cone. 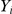 represents the binary activity of factor Y in the growth cone of neurite *i* (1 for the on state and 0 for the off state of factor Y). 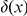 is a delta function in which 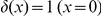 or 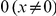. Once the on state is realized, growth continues robustly due to hysteresis (**Figure 2B**). Candidates for factor X and factor Y are discussed later (see **Discussion**).

### Local activation

Initially, each neurite is short (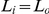), such that almost all of the factor X that is transported to the growth cone can rapidly diffuse back to the soma. Thus, the third term of Equation (1) can be dropped. The concentration of factor X in the soma increases according to 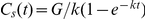 with a relatively large time constant of degradation, resulting in abundant transport to and accumulation of factor X in the growth cones. The mean concentration in the growth cone is calculated as follows (**Material and Methods 1**):
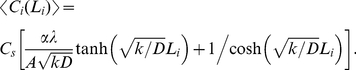
(4)


**Figure 3:**
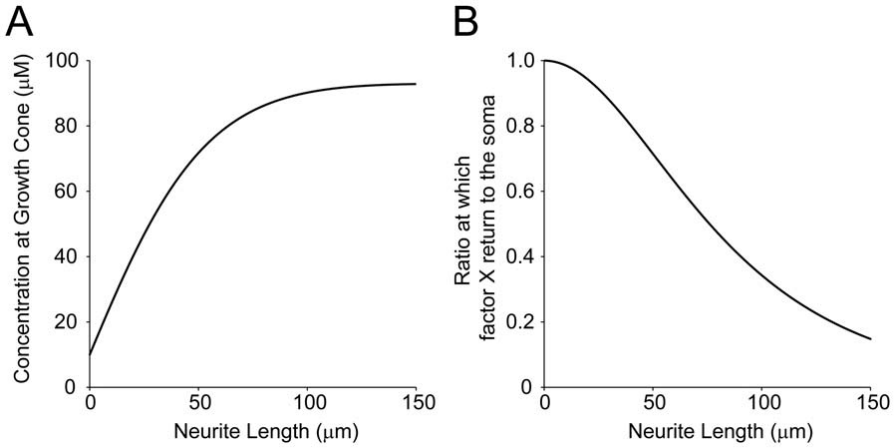
Local activation-global inhibition. (**A**) Relationship between neurite length and factor X concentration at the growth cone at steady state. This curve is mathematically obtained by Equation (4). (**B**) Relationship between neurite length and the proportion of factor X diffusing back to the soma without being degraded. This curve is mathematically obtained by Equation (5).

The concentration distribution along the neurite can be assumed to be in steady state and tied to the somatic concentration 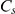 because the dynamics are dominated by rapid diffusion, compared with the slow rate of degradation within the soma. Eventually, the concentration of factor X, 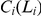, in one of the growth cones reaches a threshold *θ* (**Figure 2B**) because the time necessary to reach the threshold *θ* varies among the different growth cones. The variations in time are due to the inherent stochasticity of the transport process [3], [16], [24]. Once the threshold *θ* is reached, factor Y changes from the off state to the on state, and the corresponding neurite begins to elongate. According to Equation (4), as the neurite elongates, factor X accumulates at the neurite tip (**Figure 3A**) under the condition that the transport is sufficiently strong. This property suggests a local activation (local positive feedback) mechanism because elongation of a neurite leads to a greater accumulation of factor X, which stably maintains the activity of factor Y to facilitate further elongation of the neurite.

### Global inhibition

In long neurites, most of the transported factor X cannot return to the soma because the retrograde diffusion back takes so long time that the factor is degraded along the way. The proportion of factor X that returns to the soma is calculated as follows: 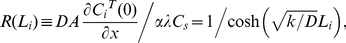
(5)where 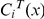 is the distribution due to transport at the neurite tip (**Material and Methods 1**). This proportion was calculated as the diffusion influx rate into the soma at the neck of the neurite divided by the transportation rate. As *L_i_* increases, *R* substantially decreases (**Figure 3B**), even if there is little degradation. This result indicates a global inhibition (global negative feedback) mechanism. With such a mechanism, a decrease in *R* caused by neurite elongation induces a decrease in the somatic concentration, resulting in a decrease in the amount of factor X transported to all the neurites. The equilibrium concentration in the soma, which depends on the length of the neurites, was approximately calculated as (**Material and Methods 2**):
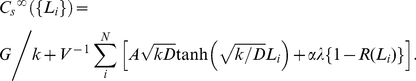
(6)


### Logic of neuronal polarization

**Figure 4:**
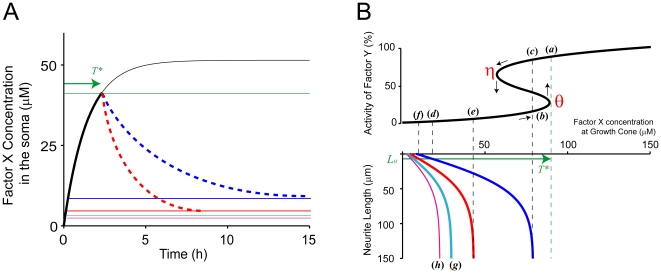
Logic of Neuronal Polarization. (**A**) Expected time course of the somatic concentration of factor X during the establishment of polarization. The black line shows the time course when no neurites are elongated, mathematically obtained as solutions to Equation (9). The expected time courses with one and two elongated neurites are depicted by the blue and red dashed curves, respectively. The solid blue, red, cyan and purple lines depict the equilibrium concentrations when one, two, three and four neurites, respectively, elongate (100 µm), as obtained from Equation (6). The green line indicates the threshold at which factor Y is activated to the on state in the growth cone of a neurite of length *L_o_*. (**B**) Activity of factor Y (upper panel) and concentration of factor X (lower panel) in the growth cone. The blue, red, cyan and purple lines in the lower panel indicate the concentration of factor X given as a function of neurite length when the somatic concentration is in equilibrium, as depicted by the solid blue, red, cyan and purple lines in (A), respectively. Points (*a* **–** *f*) indicate states when the concentration of factor X in growth cones is above (*a*) or below (*b*) the threshold *θ*, when the somatic concentration is in equilibrium as depicted by the solid blue (*c*, *d*) and red (*e*, *f*) lines in (A) and when the neurite length is infinite (*c*, *e*) and *L_o_* (*d*, *f*). The green arrows in (A) and (B) indicate the time that elapses until the concentration of factor X in one growth cone reaches the threshold *θ*. The parameters used in this analysis were 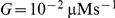, 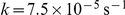, 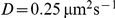, 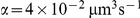, 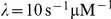, 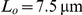, 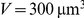, 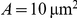, 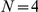 and 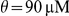. A neurite was assumed to elongate to 100 µm if factor Y switches to the on state in the corresponding growth cone.

From the mathematical analyses, we obtained a scenario of spontaneous neuronal polarization based on the phase diagram in **Figure 4**. First, the somatic concentration of factor X increases due to gene expression (black line in **Figure 4A**). The concentration of factor X in the growth cones increases according to Equation (4). The concentration in only one of the neurites happens to reach the threshold *θ* due to stochastic variations in transport. In that specific growth cone, factor Y switches from the off state to the on state, and the neurite elongates (*a* in **Figure 4B**). The concentration of factor X in the remaining neurites remains below the threshold *θ* (*b* in **Figure 4B**). The somatic concentration then starts to decrease to the level given by Equation (6) (solid blue line in **Figure 4A**), which decreases the concentration of factor X in the growth cone of the elongating neurite, but factor Y remains in the on state due to hysteresis (*c* in **Figure 4B**). The concentration of factor X in the remaining growth cones also decreases (*d* in **Figure 4B**), suggesting that the concentration of factor X in these growth cones will never reach the threshold *θ*; thereby stabilizing the disruption of symmetry (**Figure 1B**).

**Figure 5:**
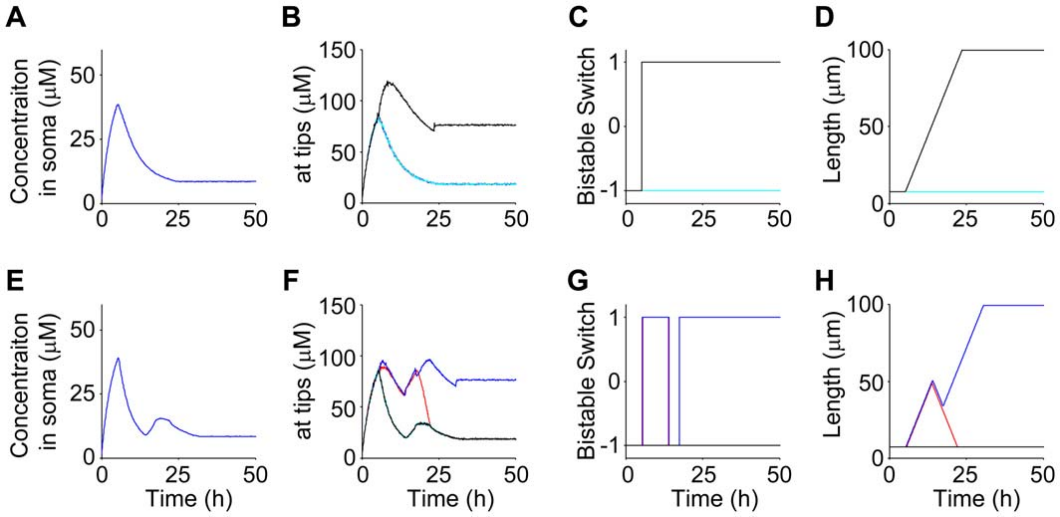
Computer simulations. The biophysical model is simulated under single-axon conditions, i.e., the threshold *η* is between the points indicated by (*c*) and (*e*) in Figure 4 (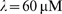). The rates of neurite growth and shrinkage are set to 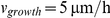 and 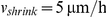, respectively. (**A–D**) Typical simulation results showing a direct transition from an initial state to a single-axon state, depicted as a transition from state (A) to state (B) in Figure 1. (**E–H**) Typical simulation result showing an indirect transition from an initial state to a single-axon state via a two-axon state, depicted as a transition from state (A) to state (B) via state (C) in Figure 1. Time courses of the concentration of factor X in the soma (**A, E**), at growth cones (**B, F**), state of factor Y (**C, G**) and neurite lengths (**D, H**) are shown. Note that four curves with different colors are plotted in (B**–**D, F**–**H) to correspond to four neurites, although distinguishing them in some cases is difficult. The other parameters used in this simulation were the same as those in Figure 4.

To validate such a scenario, we performed Monte Carlo simulations of the stochastic transport process (see **Text S1**) and successfully reproduced the expected behaviors in most trials (**Figures 5A–D**). In some trials, two neurites simultaneously elongate, as suggested by **Figure 1B**, because the concentrations in the two growth cones almost coincidentally reach the threshold *θ* (**Figures 5E–H**). After a certain period of time, one of these two neurites shrinks so that the other neurite remains to develop into an axon, indicating that favorable morphology with a single axon is flexibly sought (**Figure S8A**).

To understand the logic of this flexibility, we show that the multiple-axon state is unstable according to our phase diagram (**Figure 4**). When two neurites elongate, the somatic concentration decreases to a lower level than when a single neurite elongates (red solid line in **Figure 4A**). The factor X concentration in the growth cones of the long neurites likely falls below the threshold *η* (*e* in **Figure 4B**). Consequently, factor Y easily returns from the on state to the off state, causing the neurites to shrink to length *L_o_*. Thus, the automatic selection of a single future axon among multiple elongating neurites occurs flexibly by means of the regularization of the somatic pool of factor X. Based on the phase diagram in **Figure 4B**, we see that robust neuronal polarization is realized when the threshold *η* is between the points indicated by (*c*) and (*e*). When the threshold *η* is lower than the point indicated by (*e*), multiple axons can develop (**Figures S2** **, S3 and S8B–C**).

## Discussion

Based on a mathematical analysis and simulations of a biophysical model, we propose a mechanism of local activation-global inhibition by means of morphological changes and active transport in the absence of a global inhibitor molecule, and we show the possible logic of how a neuron autonomously searches for favorable morphology with a single axon. Our model is based on only two factors, X and Y. Factor X is only produced in the soma, and it can diffuse, be degraded (inactivated) or be actively transported. Factor Y functions as a bistable switch for axon specification. However, the real mechanism must be more complicated than our model assumptions because many molecules are involved in neuronal polarization [2] and growth cones show stochastic motility. Although some molecular details and stochastic growth cone motility were neglected, the minimal model that we used was very informative in understanding the system-level properties of neuronal polarization.

The morphology of neurons and other kinds of cells is regulated by the active transport of proteins and chemical reactions at the cellular periphery [25], [26]. We believe that our model provides a general framework for understanding the polarization of various cell types. Our model can be extended from one-dimensional neurites to two-dimensional cellular morphology, thereby expanding our knowledge beyond the easily addressable structure of neurons modeled as a round soma with one-dimensional neurites.

Several experimental studies have shown that inositol phospholipid signaling and small GTPases are involved in a positive feedback loop during axon specification [27], [28], [29], [30]. Therefore, one possible candidate for factor Y is PIP3. Factor X may be the PIP3 inducer, PI3K or a PI3K-activating factor such as HRas [15], [28], Rap1B (Ras-related protein1B) [30], Crmp2 (collapsing response mediator protein 2) [31], [32], Shootin1 [16], [19], Grb2 [33] or NudE-like (NUDEL) [34]. One possible candidate for factor X, HRas, is significantly activated only in the axonal growth cone and not in shorter neurites [15]. This study also showed that there is a mutual activation of HRas and PI3K, which is required to configure the bifurcation diagram with hysteresis as shown in **Figure 2B**. Axonal elongation is induced by the local application of actin depolymerizing reagents to neurite tips, suggesting that factor Y regulates axon specification via actin depolymerization [1]. In **Text S1**, we show a possible molecular mechanism that fits with the bifurcation diagram.

Our model can explain a number of experimental observations that are reproduced by simulations:

1. Inhibition of the ubiquitin-proteasome system induces the localization of Akt, a protein downstream of PIP3 (a factor Y candidate) in all of the neurite tips and causes multiple axons [17]. If the degradation rate *k* is low, the simulation reproduces this phenomenon with no selected neurite (**Figure S4A–D and S8D**). When *k* is small, most of the transported factor X returns to the soma, preventing significant decreases in the somatic concentration. Therefore, the winning neurite cannot easily suppress the elongation of other neurites. All of the existing models [14], [15], [16] could not explain this experimental observation, because they did not include degradation of transported molecules.
2. The motor protein kinesin-1, comprised of kinesin heavy chains (KIF5) and kinesin light chains (KLC), regulates active transport. Introducing a dominant-negative KIF5 or depleting KLC by RNAi prevents axon formation [6]. If the transport rate *λ* is low in the simulation, no neurite is selected as an axon (**Figures S5A-D and S8F**). As Equation (4) indicates, a low transport rate makes the concentration of factor X lower than the threshold *θ* in all of the growth cones, thus preventing axon formation. Our simulation predicts that polarization with a single axon is robustly achieved while increasing the transport rate *λ* (**Figures S5E–H and S8G**). The concentration of factor X at the tips (Equation (4)) is almost independent of *λ*, and this contributes to the robustness. The bracketed term in Equation (4) is almost proportional to *λ*, whereas the somatic concentration 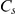 is almost inversely proportional to *λ*, as can be seen in Equation (6) when the transport rate is large.
3. Overexpression of the phosphate and tension homolog protein (PTEN) disrupts the establishment of polarity, whereas the inhibition of PTEN expression by siRNA increases the number of axons [35]. According to the model of inositol phospholipid signaling in **Text S1**, increasing PTEN elevates both the *θ* and *η* thresholds, whereas decreasing PTEN lowers both of the thresholds (**Figure S1C**). Simulations show that high and low PTEN levels induce the loss of axons and the development of multiple axons, respectively (**Figures S6 and S8H–I**).
4. The up- or down-regulation of factor X candidates, including HRas, Rap1B, Crmp2 and Shootin1, induces multiple axons or suppresses axonal formation, respectively [18], [26], [27], [29]. If the expression rate *G* is high (low), multiple (no) axons are selected in simulations (**Figures S7 and S8J–K**). Equations (4) and (6) indicate that the concentration of factor X at the growth cones varies proportionally to the expression rate *G*. Therefore, if factor X is up-regulated, the concentrations at all of the growth cones surpass the threshold *θ* even after axon formation, whereas if factor X is down-regulated, the concentrations at the growth cones never reach the threshold *θ*.
5. If the axon is transected to a shorter length than other neurites, the longest neurite becomes the axon. If the axon is cut but remains longer than the other neurites, the cut axon remains the axon [16], [36]. Equation (4) indicates that, as a neurite grows in length, the concentration of factor X at the corresponding growth cone increases, indicating that the factor X concentration in the longest neurite is closest to the threshold *θ*. Therefore, the longest neurite will most likely become the axon even after it is cut. Note that factor Y is assumed to be constantly present at the tip of the transected neurites.

Our model and simulations provide a theoretical basis for a wide variety of experimental observations, leading to a consistent understanding of the complicated morphological polarization of developing neurons. However, one aspect of our model is not supported by experiments. Our model predicts that the somatic concentration of factor X decreases after a single neurite is selected for elongation (**Figure 4A**); however, no studies report this behavior by candidate molecules for factor X. One reason for the absence of this result may be that existing experiments have monitored the quantity of molecules using GFP-fused protein, rather than directly measuring the molecular activity. We expect the activity of a certain molecule (e.g., NUDEL) to decrease in the soma after polarization. NUDEL is phosphorylated by Cdk5-kinase and forms a stable complex with lissencephaly-1 and 14-3-3ε. The NUDEL complex is transported by kinesin-1 into the neurites and is needed for axon formation [34]. Phosphorylated NUDEL may correspond to factor X in our model.

Finally, pattern formation, as exemplified by our model, captures a decoder, an important information processing function of biological systems. Cellular polarization transforms analog molecular concentrations into digital patterns of cellular morphology. A read-out mechanism to translate ambiguous analog molecular codes into rigid digital ones should exist, as demonstrated by the nonlinear but stable decoding system of molecules and morphology. A notable characteristic of such a decoding system is that it requires a back-and-forth searching mechanism over the digitalized space. In the case of polarization, the search is cancelled if pattern formation fails (i.e., if multiple axons are produced) (**Figure 1**). Cells appear to struggle autonomously and flexibly to search the space of possible morphologies for an optimal symmetry-breaking solution. This phenomenon reminds us of backtracking in searching algorithms in the field of computer intelligence. Thus, the robust read-out of biological coding systems may require mechanisms analogous to those in artificial systems.

## Materials and Methods

### 1. Distribution of Factor X along neurite

The Equation (2) is approximated by assuming point transport to neurite tips at 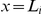, and taking a temporal average with respect to Poissonian transport variables 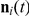,
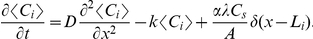



The general steady-state solution of Equation (2) can be expressed by 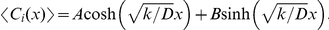



A solution with boundary conditions: 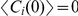 and 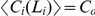 becomes 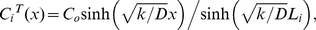
(7)which reflects the distribution due to transport to the neurite tips. *C_o_* can be determined by satisfying the detailed balance, 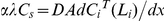. A solution with boundary conditions: 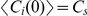 and 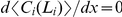 is 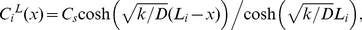
(8)which reflects the distribution due to leakage from the soma into the neurites. The sum of these solutions is the distribution realized along the neurites. Equation (4) represents the concentration at the neurite tips and can be expressed as 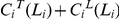.

### 2. Equilibrium concentration of factor X in the soma

To evaluate the somatic concentration dependent on neurite length, Equation (1) was approximately expressed as 
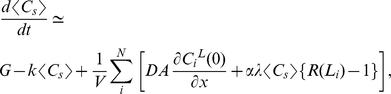



Here, we took a temporal average over the processes by which the transported factor X diffuses back to the soma using Equation (5). This equation is nothing but a simple linear differential equation:
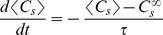
(9)with an equilibrium concentration 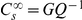 and a time constant 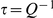, where
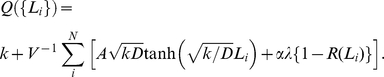



The first and second terms in the summation above represent the rate of leakage from the soma to each neurite shaft and the rate of degradation of factor X along each neurite per single unit of transport, respectively. When the neurites are kept short before polarization (*V>>NAL_o_*), these two terms can be ignored so that 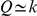. Once a neurite elongates, *Q* increases, causing the concentration of factor X in the soma to decrease.

## Supporting Information

Figure S1
**Model of the reaction network in growth cones and bifurcation diagram.** (**A**) A network model of inositol phospholipid signaling. PI3K phosphorylates PIP3, and PTEN dephosphorylates PIP2. Additionally, a positive feedback loop is mediated by HRas, a small GTPase. Feedback-regulated PI3K (PI3K*fb*) is assumed to be independent of that regulated by PI3K-activating factor. (**B**) The red and blue lines indicate the rates of phosphorylation and dephosphorylation in Equation (10), respectively, which are plotted against the PIP3 concentration. The dashed line indicates the rate of phosphorylation of PI3K regulated by the PI3K-activating factor. (**C**) This diagram depicts the PIP3 concentration at steady state when varying the PI3K regulated by the PI3K-activating factor. Parameters: 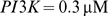, 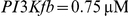, 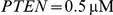, 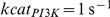, 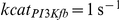, 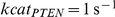, 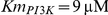, 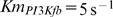, 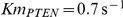, 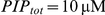, 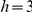 and 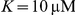.(TIF)

Figure S2
**Simulations of two-axons-possible condition.** The biophysical model simulates conditions under which two axons are possibly induced, i.e., the threshold *η* rests between the points indicated by (*e*) and (*g*) in figure 4 (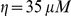). (**A–D**) Typical simulation result showing a direct transition from an initial state to a two-axon state. (**E–H**) Typical simulation result showing an indirect transition from an initial state to a two-axon state via a three-axon state. (**I–L**) Typical simulation result showing an indirect transition from an initial state to a single-axon state. Time courses for the concentration of factor X in the soma (**A, E, I**), at growth cones (**B, F, J**), the state of factor Y (**C, G, K**) and neurite length (**D, H, L**) are shown. Note that four curves with different colors are plotted in (B–D, F–H, J–L) to correspond to four neurites, although distinguishing between them is difficult in some cases.(TIF)

Figure S3
**Simulations of three-axons-possible condition.** The biophysical model simulates conditions under which three axons are possibly induced, i.e., the threshold *η* rests between the points indicated by (*e*) and (*g*) in Figure 4 (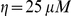). (**A–D**) Typical simulation result showing a transition from an initial state to a three-axon state. (**E–H**) Typical simulation result showing a transition from an initial state to a two-axon state. (**I–L**) Typical simulation result showing a transition from an initial state to a single-axon state. Time courses for the concentration of factor X in the soma (**A, E, I**), at growth cones (**B, F, J**), the state of factor Y (**C, G, K**) and neurite length (**D, H, L**) are shown. Note that four curves with different colors are plotted in (B–D, F-H, J–L) to correspond to four neurites, although distinguishing between them may be difficult.(TIF)

Figure S4 **Simulations of different degradation rates of factor X.** (**A–D**) Typical simulation result with a low degradation rate *k*. The *k* value here was one-fifth of the degradation rate in the standard setting (Figure 5). (**E–H**) Typical simulation result with a high degradation rate *k*. The *k* value here was five times larger than the rate in the standard setting (Figure 5). Time courses of the concentration of factor X in the soma (**A, E**), at the growth cones (**B, F**), the state of factor Y (**C, G**) and neurite length (**D, H**) are shown. Note that four curves with different colors are plotted in (B–D, F–H), to correspond to four neurites, although distinguishing between them is difficult in some cases.
(TIF)

Figure S5 **Simulations of varying rates of transport.** (**A–D**) Typical simulation result with a low rate of transports *λ*. The *λ* value here was one-fifth of the rate in the standard setting (Figure 5). (**E–H**) Typical simulation result with a high rate of transports *λ*. The *λ* value here was five times larger than the rate in the standard setting (Figure 5). Time courses of the concentration of factor X in the soma (**A, E**), at growth cones (**B, F**), the state of factor Y (**C, G**) and neurite length (**D, H**) are shown. Note that four curves with different colors are plotted in (B–D, F–H), to correspond to four neurites, although distinguishing them is difficult in some cases.
(TIF)

Figure S6
**Simulations of varying thresholds.** (**A–D**) Typical simulation result with thresholds *θ* and *η* (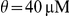, 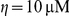) lower than those in the standard setting (Figure 5). (**E–H**) Typical simulation result with thresholds *θ* and *η* (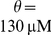, 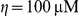) higher than those in the standard setting (Figure 5). Time courses of the concentration of factor X in the soma (**A, E**), at growth cones (**B, F**), the state of factor Y (**C, G**) and neurite length (**D, H**) are shown. Note that four curves with different colors are plotted in (B–D, F–H), to correspond to four neurites, although distinguishing between them is difficult in some cases.(TIF)

Figure S7 **Simulations of varying rates of production of factor X.** (**A–D**) Typical simulation result with a low factor X production rate *G*. The value of *G* here was one-fifth of the rate in the standard setting (Figure 5). (**E–H**) Typical simulation result with a high factor X production rate *G*. The value of *G* here was five times larger than the rate in the standard setting (Figure 5). Time courses of concentration of factor X in the soma **(A, E)**, at growth cones (**B, F**), the state of factor Y (**C, G**) and neurite length (**D, H**) are shown. Note that four curves with different colors are plotted in (B–D, F–H), to correspond to four neurites, although distinguishing between them is difficult in some cases.
(TIF)

Figure S8 **Distribution of the numbers of axons.** Each bar plot shows the distribution of the number of axons among 500 simulation runs for each condition. **(A)** Single-axon condition (Figure 4). (**B**) Two-axons possible condition (Figure S2). (**C**) Three-axons-possible condition (Figure S3). Conditions with low (**D**) and high (**E**) rates of degradation *k* for factor X (Figure S4); with low **(F)** and high (**G**) rates of transport *λ* (Figure S5); with low (**H**) and high (**I**) thresholds *θ* and *η* (Figure S6); and with low (**J**) and high (**K**) rates of production *G* of factor X (Figure S7).
(TIF)

Text S1 (DOC)
