# Supplementary figures and images for: Flexible Search for Single-Axon Morphology during Neuronal Spontaneous Polarization
Source: PLoS One. 2011 Apr 29;6(4):e19034. doi: 10.1371/journal.pone.0019034 (PMC3084731; doi:10.1371/journal.pone.0019034)

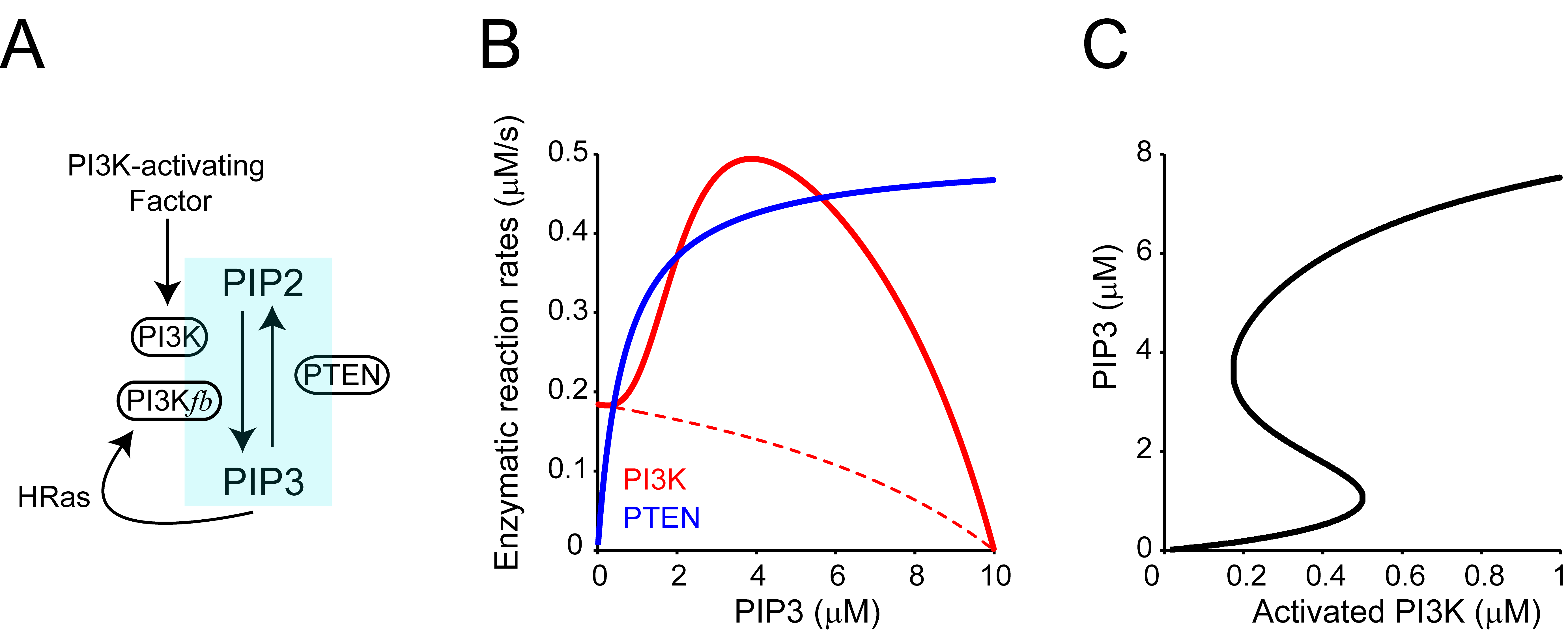

Supplement: Figure S1 — Model of the reaction network in growth cones and bifurcation diagram. (A) A network model of inositol phospholipid signaling. PI3K phosphorylates PIP3, and PTEN dephosphorylates PIP2. Additionally, a positive feedback loop is mediated by HRas, a small GTPase. Feedback-regulated PI3K (PI3Kfb) is assumed to be independent of that regulated by PI3K-activating factor. (B) The red and blue lines indicate the rates of phosphorylation and dephosphorylation in Equation (10), respectively, which are plotted against the PIP3 concentration. The dashed line indicates the rate of phosphorylation of PI3K regulated by the PI3K-activating factor. (C) This diagram depicts the PIP3 concentration at steady state when varying the PI3K regulated by the PI3K-activating factor. Parameters: , , , , , , , , , , and . (TIF) [file pone.0019034.s001.tif]

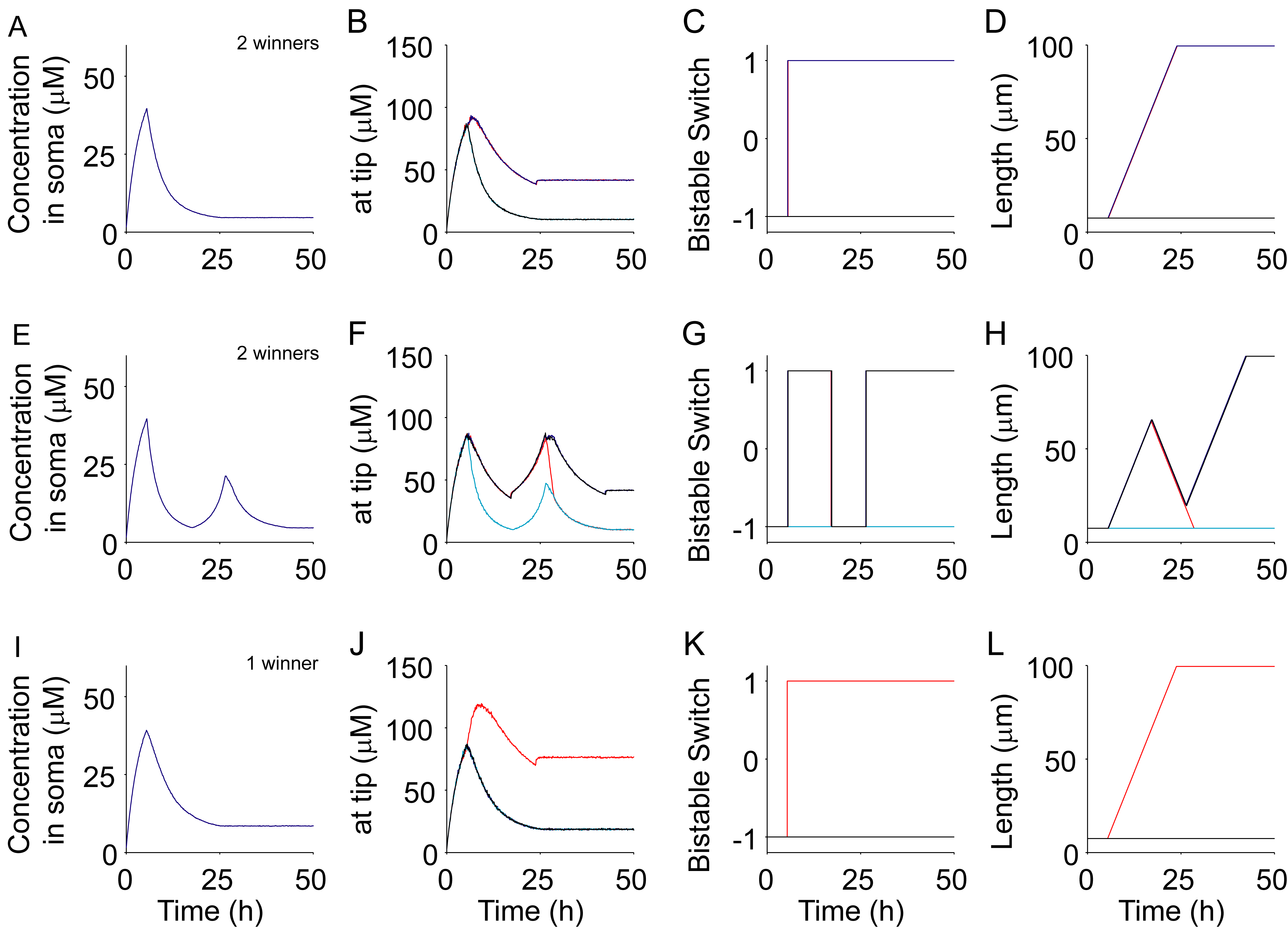

Supplement: Figure S2 — Simulations of two-axons-possible condition. The biophysical model simulates conditions under which two axons are possibly induced, i.e., the threshold η rests between the points indicated by (e) and (g) in figure 4 (). (A–D) Typical simulation result showing a direct transition from an initial state to a two-axon state. (E–H) Typical simulation result showing an indirect transition from an initial state to a two-axon state via a three-axon state. (I–L) Typical simulation result showing an indirect transition from an initial state to a single-axon state. Time courses for the concentration of factor X in the soma (A, E, I), at growth cones (B, F, J), the state of factor Y (C, G, K) and neurite length (D, H, L) are shown. Note that four curves with different colors are plotted in (B–D, F–H, J–L) to correspond to four neurites, although distinguishing between them is difficult in some cases. (TIF) [file pone.0019034.s002.tif]

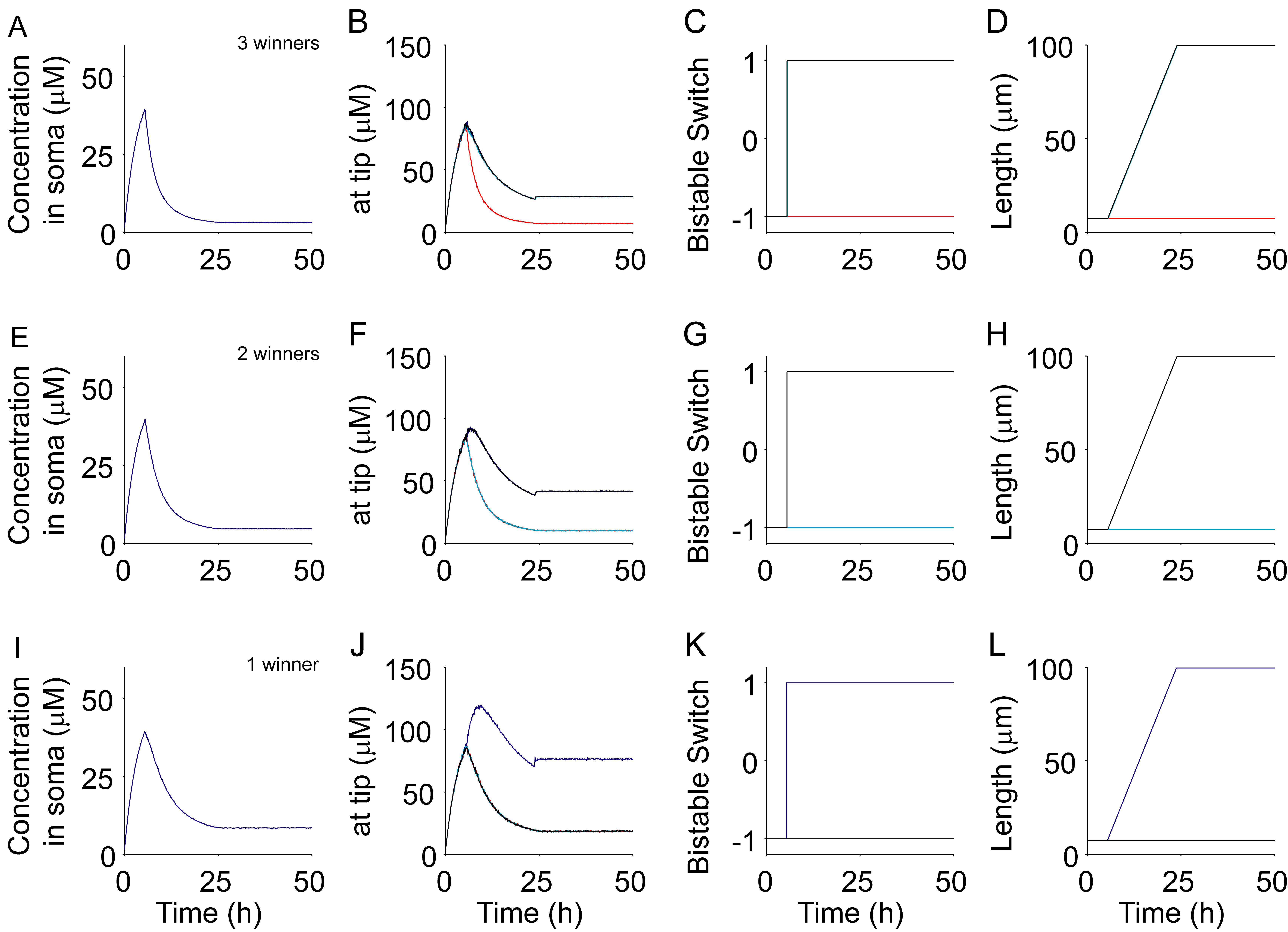

Supplement: Figure S3 — Simulations of three-axons-possible condition. The biophysical model simulates conditions under which three axons are possibly induced, i.e., the threshold η rests between the points indicated by (e) and (g) in Figure 4 (). (A–D) Typical simulation result showing a transition from an initial state to a three-axon state. (E–H) Typical simulation result showing a transition from an initial state to a two-axon state. (I–L) Typical simulation result showing a transition from an initial state to a single-axon state. Time courses for the concentration of factor X in the soma (A, E, I), at growth cones (B, F, J), the state of factor Y (C, G, K) and neurite length (D, H, L) are shown. Note that four curves with different colors are plotted in (B–D, F-H, J–L) to correspond to four neurites, although distinguishing between them may be difficult. (TIF) [file pone.0019034.s003.tif]

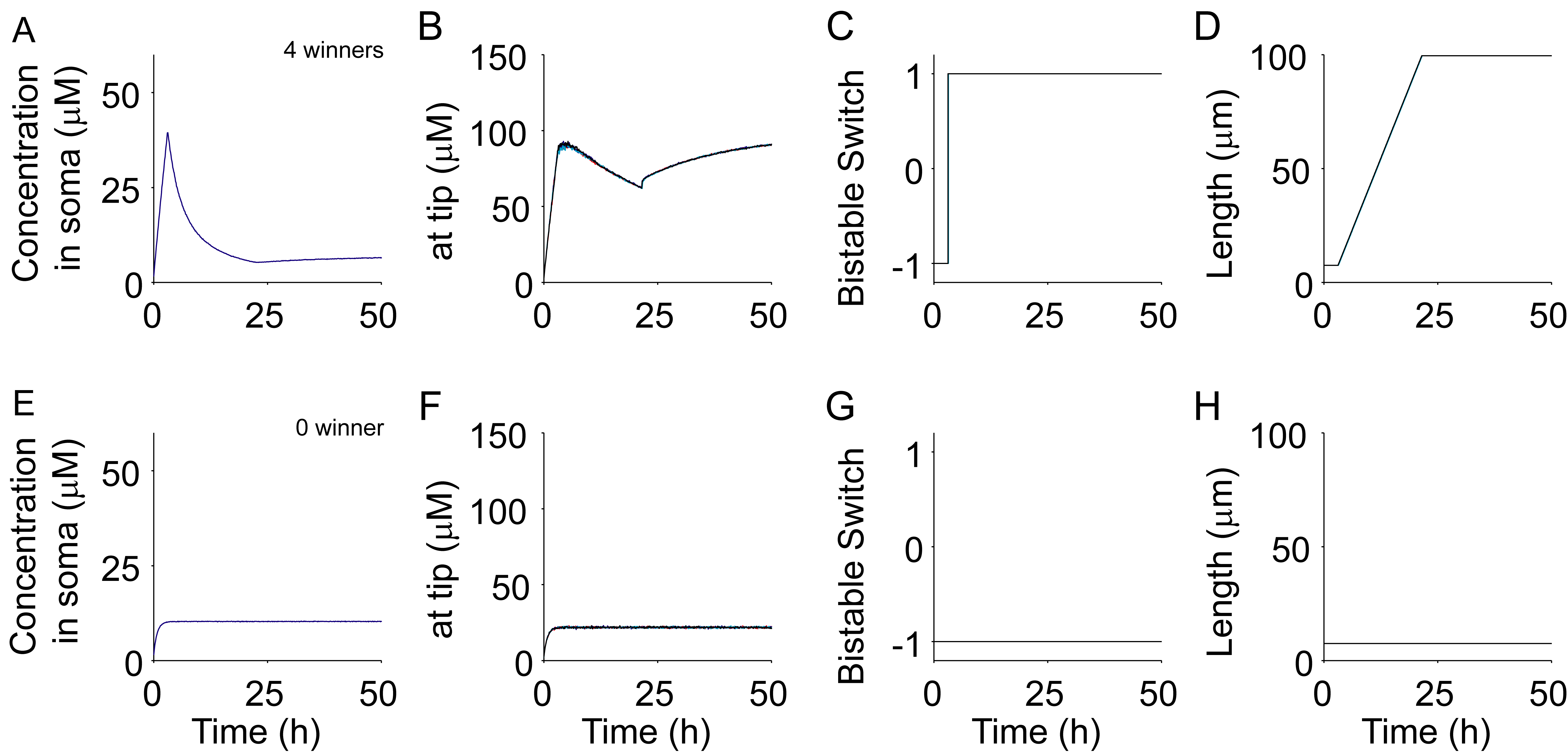

Supplement: Figure S4 — Simulations of different degradation rates of factor X. (A–D) Typical simulation result with a low degradation rate k. The k value here was one-fifth of the degradation rate in the standard setting (Figure 5). (E–H) Typical simulation result with a high degradation rate k. The k value here was five times larger than the rate in the standard setting (Figure 5). Time courses of the concentration of factor X in the soma (A, E), at the growth cones (B, F), the state of factor Y (C, G) and neurite length (D, H) are shown. Note that four curves with different colors are plotted in (B–D, F–H), to correspond to four neurites, although distinguishing between them is difficult in some cases. (TIF) [file pone.0019034.s004.tif]

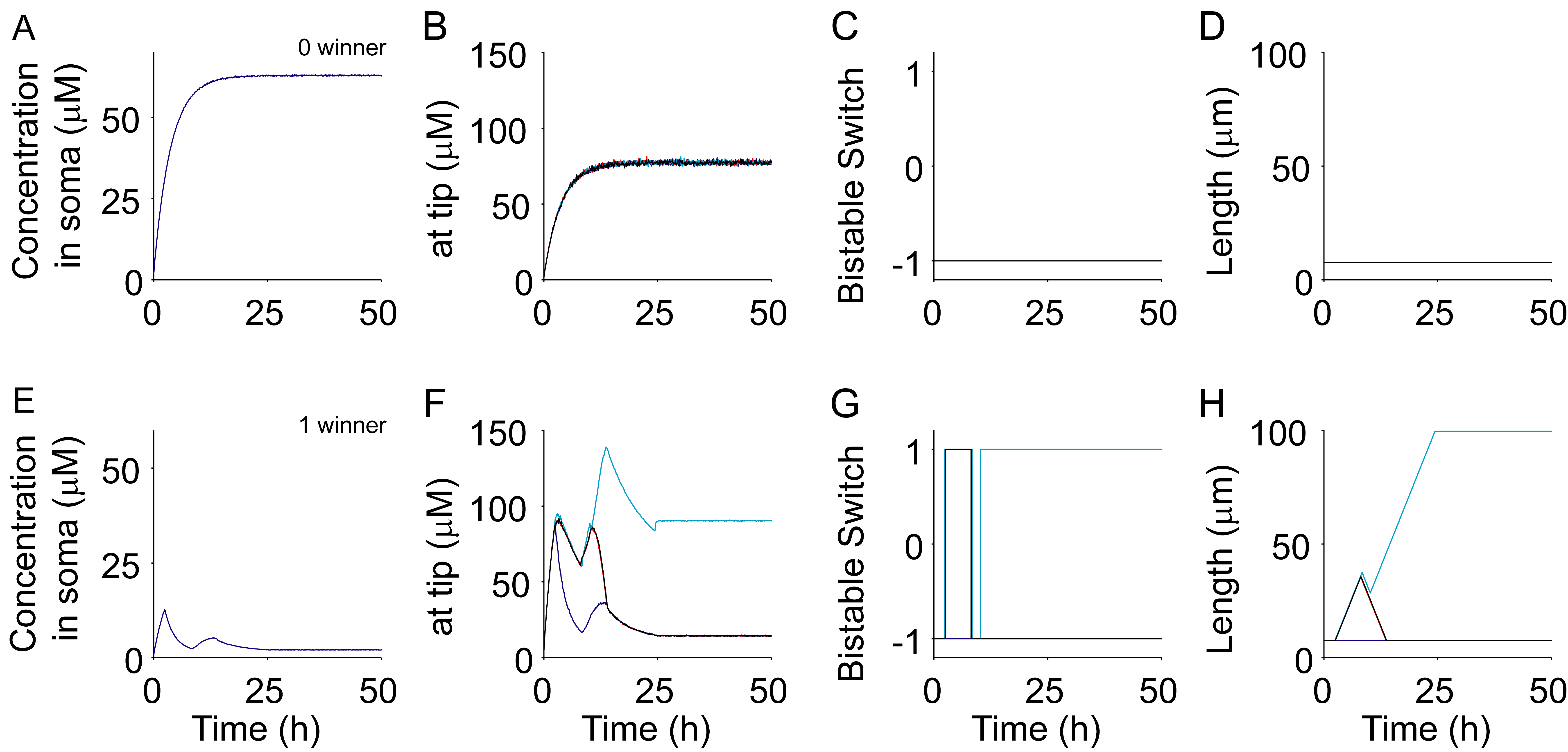

Supplement: Figure S5 — Simulations of varying rates of transport. (A–D) Typical simulation result with a low rate of transports λ. The λ value here was one-fifth of the rate in the standard setting (Figure 5). (E–H) Typical simulation result with a high rate of transports λ. The λ value here was five times larger than the rate in the standard setting (Figure 5). Time courses of the concentration of factor X in the soma (A, E), at growth cones (B, F), the state of factor Y (C, G) and neurite length (D, H) are shown. Note that four curves with different colors are plotted in (B–D, F–H), to correspond to four neurites, although distinguishing them is difficult in some cases. (TIF) [file pone.0019034.s005.tif]

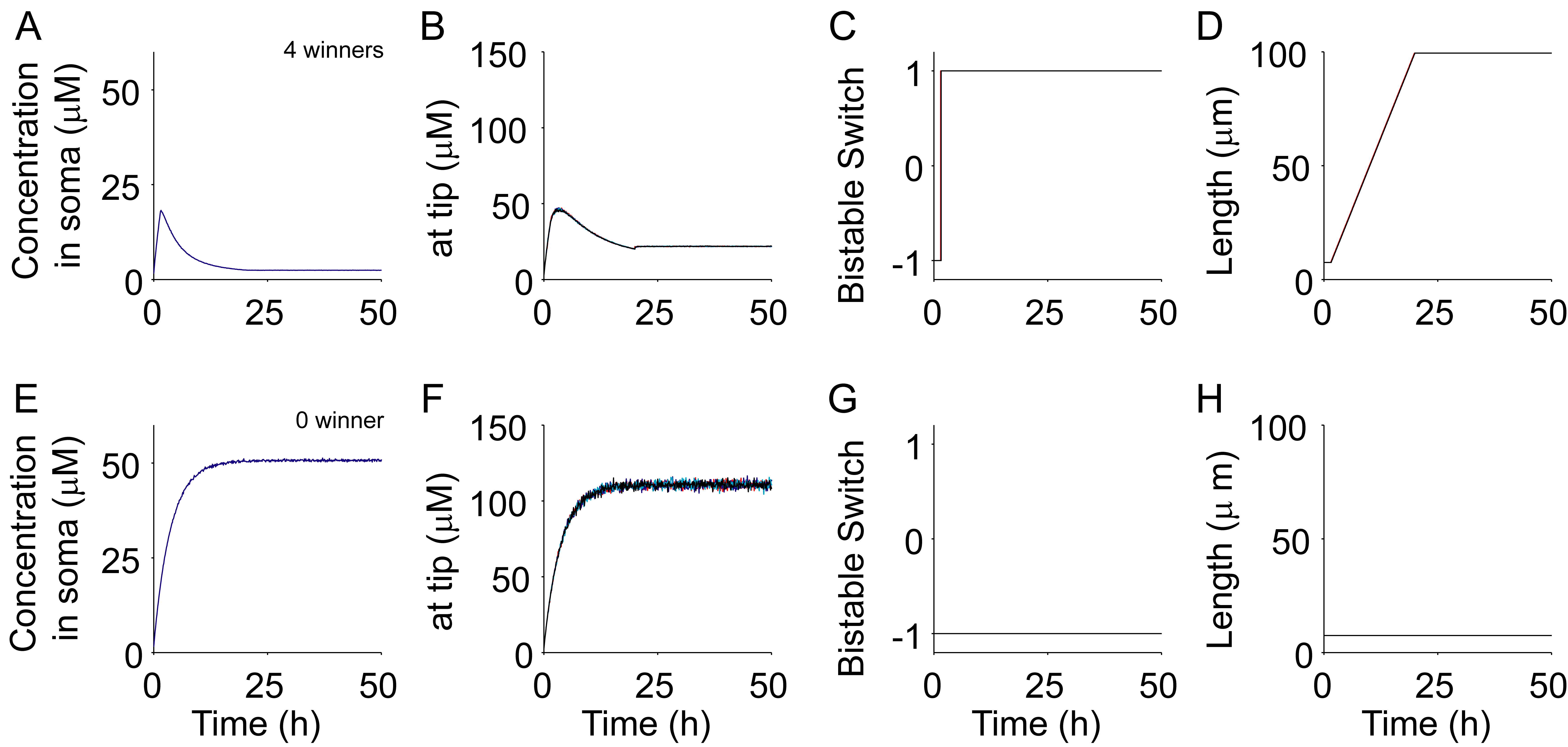

Supplement: Figure S6 — Simulations of varying thresholds. (A–D) Typical simulation result with thresholds θ and η (, ) lower than those in the standard setting (Figure 5). (E–H) Typical simulation result with thresholds θ and η (, ) higher than those in the standard setting (Figure 5). Time courses of the concentration of factor X in the soma (A, E), at growth cones (B, F), the state of factor Y (C, G) and neurite length (D, H) are shown. Note that four curves with different colors are plotted in (B–D, F–H), to correspond to four neurites, although distinguishing between them is difficult in some cases. (TIF) [file pone.0019034.s006.tif]

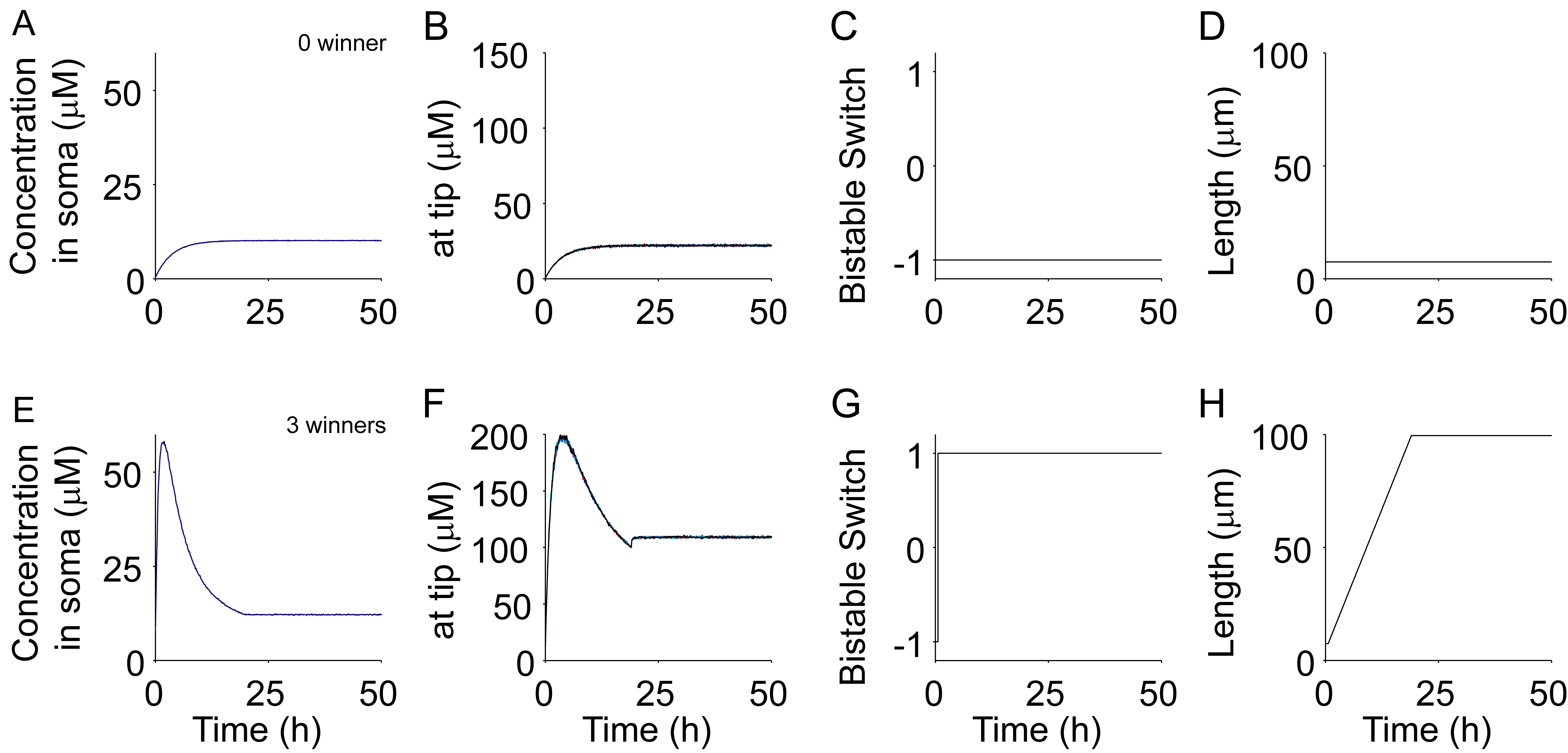

Supplement: Figure S7 — Simulations of varying rates of production of factor X. (A–D) Typical simulation result with a low factor X production rate G. The value of G here was one-fifth of the rate in the standard setting (Figure 5). (E–H) Typical simulation result with a high factor X production rate G. The value of G here was five times larger than the rate in the standard setting (Figure 5). Time courses of concentration of factor X in the soma (A, E), at growth cones (B, F), the state of factor Y (C, G) and neurite length (D, H) are shown. Note that four curves with different colors are plotted in (B–D, F–H), to correspond to four neurites, although distinguishing between them is difficult in some cases. (TIF) [file pone.0019034.s007.tif]

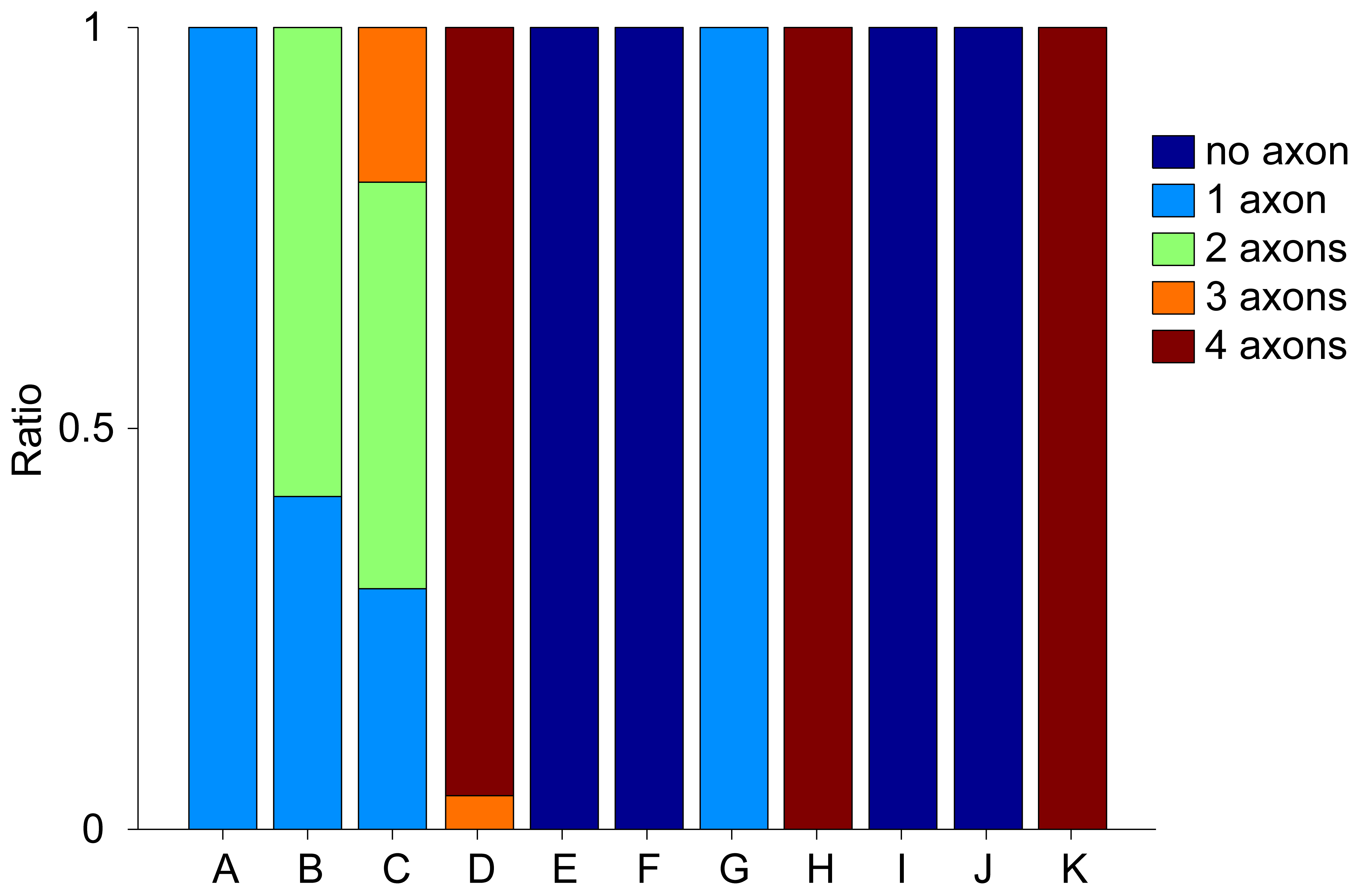

Supplement: Figure S8 — Distribution of the numbers of axons. Each bar plot shows the distribution of the number of axons among 500 simulation runs for each condition. (A) Single-axon condition (Figure 4). (B) Two-axons possible condition (Figure S2). (C) Three-axons-possible condition (Figure S3). Conditions with low (D) and high (E) rates of degradation k for factor X (Figure S4); with low (F) and high (G) rates of transport λ (Figure S5); with low (H) and high (I) thresholds θ and η (Figure S6); and with low (J) and high (K) rates of production G of factor X (Figure S7). (TIF) [file pone.0019034.s008.tif]
