# Supplementary material for: Flexible Search for Single-Axon Morphology during Neuronal Spontaneous Polarization
Source: PLoS One. 2011 Apr 29;6(4):e19034. doi: 10.1371/journal.pone.0019034 (PMC3084731; doi:10.1371/journal.pone.0019034)
Supplement: Text S1 — (DOC) [file pone.0019034.s009.doc]

**Supporting Information:**

**Flexible search for single-axon morphology during neuronal spontaneous polarization**

Honda Naoki^1^, Shinichi Nakamuta^2^, Kozo Kaibuchi^2^ and Shin Ishii^1,3^

1. Graduate School of Informatics, Kyoto University, Uji, Kyoto, Japan

2. Department of Cell Pharmacology, Nagoya University, Nagoya, Aichi, Japan

3. RIKEN Computational Science Research Program, Wako, Saitama, Japan

**Keywords**: symmetry breaking; active transport; local-activation and global-inhibition; morphogenesis; mathematical model; biophysical model; computer simulation

**Corresponding author**: Honda Naoki

**Address:** Graduate School of Informatics, Kyoto University, Gokasho, Uji, Kyoto, 611-0011, Japan

**Tel**: +81-774-38-3938

**Fax**: +81-774-38-3941

**e-mail**: n-honda@sys.i.kyoto-u.ac.jp

***Modeling biochemical reaction for axon specification***

We analyzed whether the dynamics of chemical reactions in growth cones possess a qualitative character as shown in **Figure 2B**. We developed a simple model of the reaction network in growth cones, which consists of inositol phospholipid signaling with a positive feedback loop that is supported by experimental data [1,2] (**Figure S1A**). In this model, PIP3, which is a factor Y candidate, is produced by the phosphorylation of PIP2 by PI3K and dephosphorylated by PTEN. For simplicity, we assumed that there were two types of PI3K that are independently regulated by the feedback loop and PI3K-activating factor. Here, factor X is assumed to be a PI3K-activating factor (or PI3K).

The dynamics are described by the following differential equation:

$\begin{matrix} \frac{d\text{[}PIP3\text{]}}{dt}= \frac{kcat_{PI3K} PI3K \text{[}PIP2\text{]}}{Km_{PI3K}+\text{[}PIP2\text{]}} - \frac{kcat_{PTEN} PTEN \text{[}PIP3\text{]}}{Km_{PTEN}+\text{[}PIP3\text{]}} \\ +\frac{kcat_{PI3Kfb} PI3Kfb \text{[}PIP2\text{]}}{Km_{PI3Kfb}+\text{[}PIP2\text{]}} \frac{\text{[}PIP3\text{]}^{h}}{K^{h}+\text{[}PIP3\text{]}^{h}} \end{matrix}$.

Differentiation of the PIP3 concentration [*PIP3*] consists of three terms, which are of Michaelis-Menten type formulas. $kcat_{j}$ and $Km_{j}$ $j\in\{PI3K, PI3Kfb\text{,} PTEN\}$ represent the catalytic reaction rate and the Michaelis-Menten constants, respectively. *PI3K* and *PTEN* are the concentrations of active PI3K regulated by PI3K-activating factor and PTEN, respectively, and *PI3Kfb* is the total concentration of PI3K regulated by the feedback loop. The first and the third terms represent the enzymatic reactions mediated by PI3Ks activated by PI3K-activating factor and by the feedback loop, respectively. The second term represents the reaction mediated by constitutively active PTEN. The activation ratio of feedback-regulated PI3K is determined by [*PIP3*] and is given instantaneously as a Hill equation under an equilibrium approximation that reactions in the feedback loop are rapid. *K* and *h* represent the PIP3 concentration required for half-maximal activation and the Hill coefficient, respectively. This kind of non-linearity likely comes from zero-order ultrasensitivity, which can arise from push-pull antagonistic reactions in which one enzyme activates its substrate and another enzyme inactivates it [3,4]. The positive feedback loop includes GEF and GAP, which transform GTPases (e.g., Cdc42, Rac and HRas) to their GTP- and GDP-bound forms, respectively, in an enzymatic reaction manner [5,6]. To reduce the dimensionality of the system, the total level of PIP2 and PIP3 is assumed to be constant: $PIP_{tot}=[PIP2]+[PIP3]$.

Within a wide parameter region, the phosphorylation and dephosphorylation rates in Equation , plotted against the PIP3 concentration, intersect three times, indicating a bistable structure (**Figure S1B**). A bifurcation diagram for the PIP3 concentration with varying concentrations of PI3K regulated by the PI3K-activating factor is plotted in **Figure S1C**. When the concentration is low, the system has only one stable solution in the region of low PIP3 concentration. As the concentration increases, a bifurcation appears, leading to a bistable system with two stable solutions and one unstable solution (on and off states). When the concentration increases further, another bifurcation occurs at *θ *, and the system becomes mono-stable with only one stable (on) state.

***Computer simulation***

For computer simulations, each neurite shaft was discretized into one-dimensional compartments of size *Δx*. Then, Equations (1) and (2) become spatio-temporal difference equations:

$\begin{matrix} C_{s}(t+\Delta t)=C_{s}(t)+\left[G-kC_{s}(t)+\frac{1}{V}\sum_{i}^{N} \left(DA\frac{C_{i}^{1}\text{(t)}-C_{s}(t)}{\Delta x}-\alpha C_{s}(t)\mathbf{m}_{i}(t)\right)\right]\Delta t \\ C_{i}^{n}(t+\Delta t)=C_{i}^{n}(t)+\left[D\frac{C_{i}^{n+1}(t)-2C_{i}^{n}(t)+C_{i}^{n-1}(t)}{\Delta x^{2}}-kC_{i}^{n}(t)\right]\Delta t \\ C_{i}^{tip_{i}(t+\Delta t)}(t+\Delta t)=C_{i}^{tip_{i}(t)}(t)+\left[D\frac{C_{i}^{tip(t)-1}(t)-C_{i}^{tip(t)}(t)}{\Delta x^{2}}-kC_{i}^{tip(t)}(t)\right]\Delta t+\frac{\alpha}{A\Delta x}\mathbf{m}_{i}(t) \\ C_{i}^{1}(t+\Delta t)=C_{i}^{1}(t)+\left[D\frac{C_{i}^{2}(t)-2C_{i}^{1}(t)+C_{s}(t)}{\Delta x^{2}}-kC_{i}^{1}(t)\right]\Delta t \end{matrix}$,

where $\mathbf{m}_{i}(t)$ is the number of active transport events reaching the tip of neurite *i* between times *t* and *t+Δt*; it is an independent random variable sampled from a Poisson probability distribution with frequency $\lambda C_{s}(t)\Delta t$. $C_{i}^{n}(t)$ and $tip_{i}(t)$indicate the concentration of factor X in the *n*th compartment from the neurite neck and the index number of the tip compartment, respectively.

In our simulation, factor Y within each growth cone behaves as a “flip-flop” digital switch with hysteresis, as follows (**Figure 2B**):

$Y_{i}(t+\Delta t)=\left\{\begin{matrix} 0 \\ 1 \\ Y_{i}(t) \end{matrix} \begin{matrix} if Y_{i}(t)=1 and C_{i}^{tip_{i}(t)}<\eta\\ if Y_{i}(t)=0 and C_{i}^{tip_{i}(t)}>\theta\\ else \end{matrix}\right.$.

To address the elongation and shrinkage of neurites, we introduced addition and elimination of compartments for each neurite. We heuristically assumed that there is a single resizable compartment in each neurite with a size that is dynamically controlled by the state of factor Y:

$\Delta x_{i}^{tip_{i}(t+\Delta t)-4}(t+\Delta t)=\Delta x_{i}^{tip_{i}(t)-4}(t)+\left[v_{growth}\delta(Y_{i}-1)-v_{shrink}\delta(Y_{i})\right]\Delta t$,

where $\delta(x)$ is a delta function in which $\delta(x)=1 (x=0)$ or $0 (x\neq0)$. To avoid computational instability, this resizable compartment was located at the 5th position from the neurite tip (i.e., its index is $tip_{i}(t)-4$), and its size was limited to between $\Delta x$ and $2\Delta x$. When the size of this compartment became smaller than $\Delta x$, it was fused to the 6th compartment from the tip. However, when the compartment size was larger than $2\Delta x$, it was divided into two compartments, the 6th and 5th compartments from the tip, with sizes $\Delta x$ and the remainder, respectively. With this compartment reorganization, we redistributed the molecules to fulfill the mass conservation law as follows:

$$\begin{matrix} if \Delta x<\Delta x_{i}^{tip_{i}(t+\Delta t)-4}(t+\Delta t)<2\Delta x \\ C_{i}^{tip_{i}(t+\Delta t)-4 }(t+\Delta t)=C_{i}^{tip_{i}(t)-4}(t)\Delta x_{i}^{tip_{i}(t)-4}(t)/\Delta x_{i}^{tip_{i}(t)-4}(t+\Delta t) \end{matrix}$$

$\begin{matrix} if \Delta x_{i}^{tip_{i}(t+\Delta t)-4}(t+\Delta t)<\Delta x \\ tip_{i}(t+\Delta t)=tip_{i}(t)-1 \\ \Delta x_{i}^{tip_{i}(t+\Delta t)-4}(t+\Delta t)=\Delta x_{i}^{tip_{i}(t+\Delta t)-4}(t+\Delta t)+\Delta x \\ C_{i}^{tip_{i}(t+\Delta t) to tip_{i}(t+\Delta t)-3}(t+\Delta t)=C_{i}^{tip_{i}(t) to tip_{i}(t)-3}(t) \\ C_{i}^{tip_{i}(t+\Delta t)-4 }(t+\Delta t)=\left[C_{i}^{tip_{i}(t)-4}(t)\Delta x_{i}^{tip_{i}(t)-4}(t)+C_{i}^{tip_{i}(t)-5}(t)\Delta x\right]/\Delta x_{i}^{tip_{i}(t+\Delta t)-4}(t+\Delta t) \end{matrix}$.

**References**

1. Fivaz M, Bandara S, Inoue T, Meyer T (2008) Robust neuronal symmetry breaking by Ras-triggered local positive feedback. Curr Biol 18: 44-50.

2. Arimura N, Kaibuchi K (2007) Neuronal polarity: from extracellular signals to intracellular mechanisms. Nat Rev Neurosci 8: 194-205.

3. Goldbeter A, Koshland DE, Jr. (1981) An amplified sensitivity arising from covalent modification in biological systems. Proc Natl Acad Sci U S A 78: 6840-6844.

4. Ferrell JE, Jr. (1996) Tripping the switch fantastic: how a protein kinase cascade can convert graded inputs into switch-like outputs. Trends Biochem Sci 21: 460-466.

5. Zhang B, Zheng Y (1998) Regulation of RhoA GTP hydrolysis by the GTPase-activating proteins p190, p50RhoGAP, Bcr, and 3BP-1. Biochemistry 37: 5249-5257.

6. Rudolph MG, Weise C, Mirold S, Hillenbrand B, Bader B, et al. (1999) Biochemical analysis of SopE from Salmonella typhimurium, a highly efficient guanosine nucleotide exchange factor for RhoGTPases. J Biol Chem 274: 30501-30509.
